# Surgical treatment of scoliosis in Rubinstein-Taybi syndrome type 2: a case report

**DOI:** 10.1186/1752-1947-9-10

**Published:** 2015-01-18

**Authors:** Nikolaos Bounakis, Christos Karampalis, Hilary Sharp, Athanasios I Tsirikos

**Affiliations:** Scottish National Spine Deformity Centre, Royal Hospital for Sick Children, Sciennes Road, Edinburgh, EH9 1LF UK

**Keywords:** Posterior spinal fusion, Rubinstein-Taybi syndrome, Scoliosis, Surgical treatment

## Abstract

**Introduction:**

Rubinstein-Taybi syndrome is an autosomal dominant disorder resulting in congenital craniofacial deformities, and divided into types 1 and 2. Scoliosis has not been reported as one of the extra-cranial manifestations of Rubinstein-Taybi syndrome type 2.

**Case presentation:**

We present a 14-year-old British Caucasian girl with Rubinstein-Taybi type 2 syndrome who developed a severe double thoracic scoliosis measuring 39° and 68° respectively. Her scoliosis was associated with thoracic hypokyphosis, causing a marked reduction in the anteroposterior diameter of her chest and consequent severe restrictive lung disease. The deformity was noted by her local pediatrician as part of a chest infection assessment when she was aged 13 years, and gradually progressed as the result of spinal growth. Our patient underwent a posterior spinal arthrodesis using a single concave pedicle hook and screw rod construct and locally harvested autologous graft supplemented by allograft bone. This spinal fixation technique was selected because of our patient’s low body weight to avoid prominence of the instrumentation causing skin healing problems and pain. Her scoliosis was corrected to 18° and 30° and we achieved a balanced spine in the coronal and sagittal planes. An underarm spinal jacket was provided for six months after surgery. During her latest follow-up at skeletal maturity, our patient had an excellent cosmetic outcome with no loss of deformity correction or detected pseudoarthrosis and a normal level of activities.

**Conclusion:**

Scoliosis can develop in young children with Rubinstein-Taybi syndrome type 2, with the deformity deteriorating around the pubertal growth spurt. Surgical treatment can correct the deformity, balance the spine and prevent mechanical back pain. It can also stabilize the chest area and avoid respiratory complications developing as the scoliosis progresses, which can result in severe restrictive pulmonary disease. The use of single concave instrumentation is indicated in very slim patients with poor muscle bulk; in our patient, this produced satisfactory deformity correction and a favorable outcome at completion of growth. Peri-operative care in this group of patients can be very challenging because of associated co-morbidities as well as the presence of severe behavioral issues that result in poor patient compliance.

## Introduction

Rubinstein-Taybi syndrome (RTS) is an autosomal dominant disorder resulting in craniofacial dysmorphism with distinctive facial features, short stature, developmental delay, and mental retardation. Spinal deformity and other orthopedic manifestations have been described as primary features of RTS type 1 (RTS-1). We present the case of a patient with RTS-2 who developed severe double thoracic scoliosis and underwent a posterior spinal arthrodesis. We describe our patient’s post-operative course and final outcome 2.2 years after scoliosis surgery, when the patient had completed her skeletal growth. To the best of our knowledge, this is the first report of a patient with RTS-2 developing scoliosis and requiring surgical treatment.

## Case presentation

A British Caucasian girl aged 14 years was referred to our clinic with a double thoracic scoliosis. She was diagnosed with RTS-2 on the basis of clinical findings and genetic testing. A physical examination demonstrated down-slanting palpebral fissures, bilateral proptosis, divergent squint right eye, micrognathia and microcephaly, a high arched and narrow palate, dental crowding, ears posteriorly rotated, and arachnodactyly. As part of the underlying condition, our patient had marked nasal speech and mild to moderate right conductive hearing impairment. She had developmental delay and attention deficit hyperactivity disorder. She had a learning disability (intelligence quotient (IQ) 60) and delay in fine motor skills. There was no history of recurrent chest infections or gastroesophageal reflux. There was no family history of syndromic conditions or scoliosis. The scoliosis was first noted at the age of 13 years during an assessment of a chest infection by her pediatrician. No treatment was given at that stage and the deformity gradually progressed. At presentation to our clinic, she was post-menarche with height 137cm, arm span 144cm, body weight 27kg, and body mass index 9.8kg/m^2^.

On clinical examination, our patient had a severe double left upper and right lower thoracic scoliosis. Her thoracic spine across the more severe right thoracic curve was rotated to the right, with marked ipsilateral prominence of her rib cage and scapula and associated hypokyphosis. There was also thoracic translocation and listing of her trunk to the right, with associated waistline asymmetry and prominence of the left side of her pelvis. Her pelvis was level with no evidence of leg-length discrepancy. There were no skin or soft tissue abnormalities overlying her spine. Our patient reported no neurological symptoms. A neurological examination confirmed normal tone, muscle power, sensation and tendon reflexes in her upper and lower limbs, as well as symmetrically elicited abdominal reflexes. There were no upper motor neuron signs. Moderate ligamentous laxity was noted.

Radiographs of her spine during the initial assessment revealed a right thoracic scoliosis extending from T6 to L2, measuring 46°, and a left upper thoracic scoliosis extending from T1 to T6, measuring 30°. The rotatory component of the deformity resulted in marked thoracic hypokyphosis, which in turn significantly reduced the anteroposterior diameter of her chest, as well as the space available for her lungs. There were no congenital anomalies affecting her vertebral column or chest wall. There were also no features suggestive of congenital spinal stenosis, with the interpedicular distance within normal limits across all spinal segments. Her Risser grade was 0 with open triradiate cartilage bilaterally, indicating that she had significant remaining skeletal growth.

Because of the severity of her scoliosis, we decided to proceed with surgical correction. In the presence of the underlying syndromic condition, a pre-operative assessment was organized that included spinal magnetic resonance imaging (MRI) under general anesthesia as well as cardiac; anesthetic; ear, nose and throat; psychological; and respiratory reviews. We encountered extreme difficulty in concluding the pre-operative assessment because our patient’s anxiety, behavioral problems and poor cooperation resulted in severe delays.

The MRI of the spine demonstrated no intra-spinal anomalies, normal appearance of the pedicles and no spinal stenosis. There was no evidence of tracheal or bronchial obstruction. The psychological evaluation concluded that our patient was cognitively and emotionally younger than her chronological age and consistent with that of a child aged seven to eight years. The cardiac evaluation, including electrocardiogram and ultrasound, showed normal function. The respiratory review, including chest radiographs, capillary blood gas sample and sleep studies, demonstrated restrictive pulmonary disease with marked deterioration of lung functions, forced expiratory volume in one second (FEV_1_) 0.79L (38% predicted) and forced vital capacity (FVC) 0.88L (37% predicted). Pre-operative blood results were within normal limits. At the time of surgery, 10 months after her initial clinical presentation, the degree of scoliosis in the thoracic curves had progressed to 39° and 68° respectively. Both curvatures were significantly rigid on a supine maximum traction radiograph, with the flexibility index being 7% and 20% for the upper and lower thoracic scoliosis respectively.

**Figure 1:**
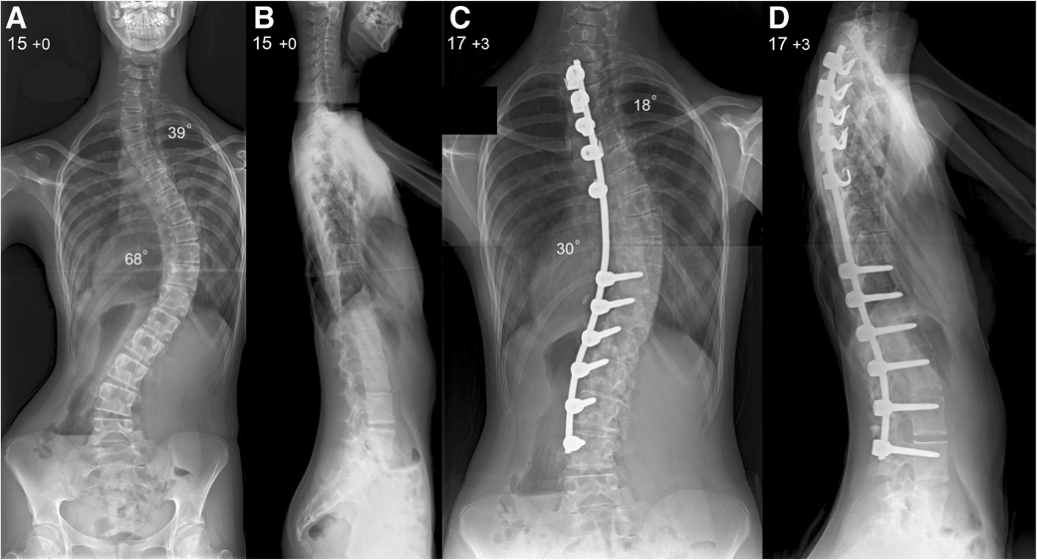
**Pre-operative** **(A)** **postero-anterior and** **(B)** **lateral radiographs of the spine show a very severe double thoracic scoliosis producing thoracic translocation and spinal decompensation to the right with a marked ipsilateral rib hump and waistline asymmetry.** **(C)** Postero-anterior and **(D)** lateral radiographs of the spine at latest follow-up show excellent scoliosis correction across both thoracic curves and an overall balanced spine in the coronal and sagittal planes.

Our patient was admitted to our hospital on the day of surgery and the plan was for her to receive a dose of midazolam as pre-medication before transfer to theater. However, she refused to take her pre-medication or go to theater, despite efforts and involvement of allied health specialists (specialist spinal nurse, learning disability nurse and health play specialist). This resulted in cancellation of the procedure. Additional psychological support sessions were offered to our patient and the surgery was re-scheduled with a different anesthetic plan. A peripheral intravenous access was secured when performing pre-operative blood tests that allowed us to administer propofol and remifentanil in the Surgical Admissions Unit. She was subsequently transported to theater with an oxygen face mask, where intubation was performed without complications. There were no difficulties during intubation related to our patient’s craniofacial anomalies.Our patient (now 15 years old) underwent a posterior spinal arthrodesis extending from T2 to L4 with the use of a pedicle hook, screw and single concave rod instrumentation (Figure 1A,B). We performed subperiosteal exposure of the spine to the tips of the transverse processes with extensive facetectomies to mobilize the curve and allow for placement of the instrumentation and extensive decortication. This was followed by an interfacetal and intertransverse arthrodesis using locally harvested bone from the spinous processes and supplemented by allograft bone. Correction of the scoliosis was achieved through apical translation, rod de-rotation, and proximal or distal distraction of the construct. We monitored her spinal cord during the operation, recording cortical and cervical somatosensory as well as transcranial motor evoked potentials, and there were no problems. A nasogastric tube was placed at completion of surgery to allow early instigation of feedings. Our patient was transferred to our intensive care unit still intubated.Our patient was extubated on her first post-operative day and remained in our intensive care unit for two days. Feedings were achieved through a nasogastric tube with the addition of nutritional supplements. An underarm spinal jacket was applied for six months after surgery to provide additional support. Her total hospital stay was 17 days and overnight nasogastric feedings were continued at home. Post-operative radiographs showed satisfactory correction of both thoracic curves to 18° and 30° respectively and restoration of the sagittal balance of her spine (Figure 1C,D).

At her latest follow-up, two years and three months after surgery, our patient had no complaints of her back and she had returned to her normal activities. She was skeletally mature and spinal radiographs demonstrated no loss of scoliosis correction, no non-union and no add-on junctional deformity above or below the levels of the fusion. Repeat pulmonary function tests demonstrated a mild improvement in her lung function compared with her pre-operative tests, with FEV_1_ of 0.86L (47% predicted) and FVC of 0.94L (49% predicted).

## Discussion

RTS is a rare congenital multi-system disorder characterized by mental retardation, post-natal growth deficiency, microcephaly, broad thumbs and halluces, and dysmorphic facial features. The condition was first described in 1957 by Michail *et al*. [1] in a French orthopedic journal. In 1963, Rubinstein and Taybi described a malformation syndrome characterized by distinctive facial features, mental deficiency, and broad thumbs and halluces in seven patients [2, 3]. The following year, Coffin [4] reported an additional six cases and proposed the eponymous title. RTS is alternatively known as broad thumb and hallux syndrome.

The condition has a variable degree of phenotypic expression. There are two distinct types: type 1 is caused by a heterozygous mutation in the gene encoding the transcriptional co-activator CREB-binding protein (CREBBP) on chromosome 16p13 [5], and type 2 by a *de novo* heterozygous mutation in the EP300 gene on chromosome 22q13 [6]. Our patient had type 2 disorder (deletion c.494-497delTgAA in exon 2 of the EP300 gene). CREBBP is a transcriptional co-activator in cyclic adenosine monophosphate-mediated intracellular protein synthesis and as such is an important regulator of cell growth and division. CREBBP is also a critical co-activator for thyroid hormone receptors [7] essential for normal fetal development, central nervous system development and possibly memory.

RTS is uncommon and affects one in 100,000 to 125,000 neonates [8]. Both type 1 and 2 are considered to have an autosomal dominant pattern of inheritance [6]. A diagnosis of RTS is essentially clinical because the characteristic cytogenetic or molecular abnormality (mutations in either CREBBP or p300 or microdeletion at 16p13.3) can be detected only in 55% of patients, leaving the diagnosis in the remaining 45% to be based on clinical features alone. Prenatal diagnosis is possible by DNA analysis extracted from fetal cells obtained by amniocentesis (15 to 18 weeks’ gestation) or chorionic villus sampling (10 to 12 weeks’ gestation). Prenatal sonography is useful in the detection of physical fetal anomalies as an adjunct to genetic screening [9].

The striking facial features and characteristic hand and foot findings of RTS mean that it is frequently recognized at birth or in infancy. Typical facial characteristics include a beaked nose, broad nasal bridge, cleft palate anomaly, grimacing smile, palpebral fissures, anti-mongoloid slant, apparent hypertelorism, coloboma of the eyes and the lower eyelids with a paucity of eyelashes [2–4]. Head circumference under the 50th percentile (95%), microcephaly (87%), strabismus (60,7%), refractive errors (50%) and cataract are also common. The ears are often abnormal in position, rotation, size or shape (75.7%) with atresia of the external auditory canals and bilateral conductive hearing loss. Patients with RTS have an increased risk of malignancies, affecting mainly the central nervous (oligodendroglioma, medulloblastoma, neuroblastoma, meningioma) and hematological systems (leukemia and lymphoma) [10]. Congenital cardiac anomalies can also occur in 24% to 38% of patients [11].

The most characteristic finding is broad terminal phalanges in the thumbs and first toes (99%); other fingers may have a similar appearance (74%). Brisk reflexes have been observed. Hypotonia, ligamentous laxity and hyper-extensible joints are also very frequent (71.6%). Legg-Calve-Perthes disease (3%), dislocated patella (2.5%), congenital hip dislocation (1.4%) and slipped capital femoral epiphysis (0,6%) have been described [3, 12–18]. These features are likely to account for a typical stiff hip or knee gait with associated flat feet deformity (83.8%).

Delayed development is typical and mental, language and social retardation are common symptoms (98.5%) with the IQ often being under 50. Short attention span, impulsivity, clinically non-significant stereotypy, decreased tolerance for noise and crowds, hyperactivity, attention problems, withdrawal and nonspecific mal-adaptive behaviors such as self-injurious and aggressive have been associated with RTS. Previous reports describe a very clear psychiatric phenotype, characterized by autistic spectrum and tic disorders, major depressive disorder with obsessive-compulsive features, bipolar disorder, and psychosis [19, 20]. The behavioral disorders associated with the condition explain the severe problems that we encountered both during the pre-operative assessment towards scoliosis surgery but also in the peri-operative care, which was highly challenging.

Up to 70% of patients with RTS have type 1, whereas RTS-2 is much less common and represents a milder phenotype. Patients with RTS-2 have less severe facial dysmorphism and better cognitive function, but may have more severe microcephaly and malformation of facial bone structures compared with those with RTS-1. Spinal anomalies, such as spina bifida, congenital or acquired scoliosis, kyphosis, and lordosis have been commonly described in patients with RTS-1 (75.3%) [3, 12–14]. There have been previous reports of surgical treatment to address thumb deformity, polydactyly, patellar dislocation, cervical myelopathy and tethered cord, with no documented peri-operative complications [13–18]. The development of keloid and hypertrophic scars is a common problem in patients with RTS and should be taken into account [21].

We could find only one previous report in the literature of a patient with RTS-1 undergoing anterior and posterior spinal fusion to correct a thoracic and lumbar scoliosis with a good outcome [14]. Our patient with RTS-2 developed a severe double thoracic scoliosis that progressed significantly during puberty. She also had severe thoracic hypokyphosis resulting in a reduction in the antero-posterior diameter of her chest. The subsequent restrictive lung disease was an additional indicator for surgical correction. We elected to use a posterior approach and a hybrid (hook and screw) rod construct to stabilize her spine, correct the scoliosis across the two thoracic curves, and restore thoracic kyphosis. A single concave rod was used because our patient was severely underweight and a convex instrumentation would have been very prominent under her skin, potentially producing wound-healing problems and muscular pain. The use of an abundant bone graft followed by post-operative support through a spinal jacket allowed for a solid fusion and no recurrence of the deformity at follow-up. The initial challenging post-operative course was mainly due to poor patient compliance and difficulties re-establishing adequate feedings. At our latest follow-up, our patient had an excellent cosmetic result and a balanced spine, as well as improved respiratory function.

## Conclusion

Scoliosis has not been previously reported in children with RTS-2. The deformity is progressive during the adolescent growth spurt and can produce severe thoracic distortion and restrictive pulmonary compromise. Surgical correction can be very challenging in the presence of multiple recognized co-morbidities but also because of significant behavioral and anxiety issues that result in high patient resistance and aggressiveness. Pre-operative assessment needs to exclude associated feeding, cardiac, respiratory and upper airway abnormalities that can increase surgical morbidity. The use of a single concave rod technique may be indicated in patients who are very slim because instrumentation cover is often difficult, resulting in wound infection and post-operative pain. Adequate feedings have to be established in the immediate post-operative period to allow optimum wound healing. Scoliosis correction and restoration of normal sagittal spinal balance can improve cosmesis, prevent muscular back pain and improve restrictive lung disease while allowing for return to normal function.

## Consent

Written informed consent was obtained from the patient’s legal guardians for publication of this case report and any accompanied images. A copy of the written consent is available for review by the Editor-in-Chief of this journal.

## Abbreviations

CREBBP: CREB-binding protein
FEV_1_: forced expiratory volume in one second
FVC: forced vital capacity
MRI: magnetic resonance imaging
RTS: Rubinstein-Taybi syndrome.

## References

[1] Michail J, Matsoukas J, Theodorou S: **Arched, clubbed thumb in strong abduction-extension & other concomitant symptoms.** *Rev Chir Orthop Reparatrice Appar Mot* 1957,**43**(2)**:**142–6. 13466652

[2] Rubinstein JH, Taybi H: **Broad thumbs and toes and facial abnormalities: A possible mental retardation syndrome.** *Am J Dis Child* 1963, **105:**588–608. 10.1001/archpedi.1963.02080040590010 10.1001/archpedi.1963.02080040590010 13983033

[3] Rubinstein JH: **Broad thumb-hallux (Rubinstein-Taybi) syndrome 1957–1988.** *Am J Med Genet Suppl* 1990, **6:**3–16. 10.1002/ajmg.1320370603 2118774

[4] Coffin GS: **Brachydactyly, peculiar facies and mental retardation.** *Am J Dis Child* 1964, **108:**351–9. 10.1001/archpedi.1964.02090010353004 14186654

[5] Oike Y, Hata A, Mamiya T, Kaname T, Noda Y, Suzuki M, *et al*.: **Truncated CBP protein leads to classical Rubinstein-Taybi syndrome phenotypes in mice: implications for a dominant-negative mechanism.** *Hum Mol Genet* 1999, **8:**387–96. 10.1093/hmg/8.3.387 10.1093/hmg/8.3.387 9949198

[6] Bartsch O, Labonte J, Albrecht B, Wieczorek D, Lechno S, Zechner U, *et al*.: **Two patients with EP300 mutations and facial dysmorphism different from the classic Rubinstein-Taybi syndrome.** *Am J Med Genet* 2010, **152A:**181–4. 10.1002/ajmg.a.33153 10.1002/ajmg.a.33153 20014264

[7] Olson DP, Koenig RJ: **Thyroid function in Rubinstein-Taybi syndrome.** *J Clin Endocr Metab* 1997, **82:**3264–6. 10.1210/jcem.82.10.4273 9329350

[8] Hennekam RC, VandenBoogaard MJ, VanDoorne JM: **A cephalometric study in Rubinstein- Taybi syndrome.** *J Craniofac Genet Dev Biol* 1991, **20:**33–40. 2061404

[9] Greco E, Sglavo G, Paladini D: **Prenatal sonographic diagnosis of Rubinstein-Taybi syndrome: a case report.** *J Ultrasound Med* 2009, **28:**669–72. 10.7863/jum.2009.28.5.669 19389907

[10] Miller RW, Rubinstein JH: **Tumors in Rubinstein-Taybi syndrome.** *Am J Med Genet* 1995, **56:**112–5. 10.1002/ajmg.1320560125 10.1002/ajmg.1320560125 7747773

[11] Stevens CA, Bhakta MG: **Cardiac abnormalities in the Rubinstein-Taybi syndrome.** *Am J Med Genet* 1995, **59:**346–8. 10.1002/ajmg.1320590313 10.1002/ajmg.1320590313 8599359

[12] Tatara Y, Kawakami N, Tsuji T, Miyasaka K, Ohara T, Nohara A: **Rubinstein-Taybi syndrome with scoliosis: a case report.** *Scoliosis* 2011, **6:**21. 10.1186/1748-7161-6-21 10.1186/1748-7161-6-21 PMC3198739 21961764

[13] Tanaka T, Ling BC, Rubinstein JH, Crone KR: **Rubinstein-Taybi syndrome in children with tethered spinal cord.** *J Neurosurg* 2006, **105:**261–4. 10.3171/ped.2006.105.4.261 17328274

[14] Robson MJ, Brown LM, Sharrard WJ: **Cervical spondylolisthesis and other skeletal abnormalities in Rubinstein-Taybi syndrome.** *J Bone Joint Surg Br* 1980, **62:**297–9. 10.1302/0301-620X.62B3.7410460 7410460

[15] Bonioli E, Bellini C, Senes FM, Palmieri A, DiStadio M, Pinelli G: **Slipped capital femoral epiphysis associated with Rubinstein-Taybi syndrome.** *Clin Genet* 1993, **44:**79–81. 10.1111/j.1399-0004.1993.tb03851.x 8275564

[16] Mehlman C, Rubinstein J, Roy D: **Instability of the patellofemoral joint in Rubinstein-Taybi syndrome.** *J Pediatr Orthop* 1998, **18:**508–11. 9661863

[17] Wood VE, Rubinstein JH: **Surgical treatment of the thumb in the Rubinstein-Taybi syndrome.** *J Hand Surg Br* 1987, **12:**166–72. 10.1016/0266-7681_87_90005-2 3624969

[18] Stevens CA: **Patellar dislocation in Rubenstein-Taybi syndrome.** *Am J Med Genet* 1997, **72:**188–90. 10.1002/(SICI)1096-8628(19971017)72:2<188::AID-AJMG12>3.0.CO;2-J 9382141

[19] Hennekam RC, Baselier AC, Beyaert E: **Psychological and speech studies in Rubinstein-Taybi syndrome.** *Am J Ment Retard* 1992, **96:**645–60. 1344941

[20] Levitas A, Reid CS: **Rubinstein-Taybi syndrome and psychiatric disorders.** *J Intellect Disabil Res* 1998, **42:**284–92. 10.1046/j.1365-2788.1998.00136.x 9786443

[21] Siraganian P, Rubinstein J, Miller R: **Keloids and neoplasms in Rubinstein-Taybi syndrome.** *Med Pediatr Oncol* 1989, **17:**485–91. 10.1002/mpo.2950170526 2586363

